# Propolis in Dental Implantology: A Systematic Review of Its Effects and Benefits

**DOI:** 10.3390/jfb15110339

**Published:** 2024-11-12

**Authors:** Magdalena Sycińska-Dziarnowska, Liliana Szyszka-Sommerfeld, Magdalena Ziąbka, Krzysztof Woźniak, Gianrico Spagnuolo

**Affiliations:** 1 Department of Maxillofacial Orthopaedics and Orthodontics, Pomeranian Medical University in Szczecin, Al. Powst. Wlkp. 72, 70111 Szczecin, Poland; 2 Laboratory for Propaedeutics of Orthodontics and Facial Congenital Defects, Pomeranian Medical University in Szczecin, Al. Powst. Wlkp. 72, 70111 Szczecin, Poland; 3 Faculty of Materials Science and Ceramics, Department of Ceramics and Refractories, AGH University of Krakow, 30059 Krakow, Poland; 4 Department of Neurosciences, Reproductive and Odontostomatological Sciences, University of Naples “Federico II”, 80131 Napoli, Italy; 5 School of Dentistry, College of Dental Medicine, Kaohsiung Medical University, Kaohsiung 80708, Taiwan

**Keywords:** propolis, dental implants, antimicrobial

## Abstract

Dental implants are widely recognized for their effectiveness in restoring missing teeth, yet their success is often compromised by infections or inadequate osseointegration. Propolis, a natural resinous substance with potent antimicrobial, anti-inflammatory, and osteogenic properties, has emerged as a promising adjunct in dental implantology. This systematic review critically evaluates the current evidence on the incorporation of propolis into dental implants, focusing on its impact on antimicrobial efficacy, bone healing, and overall implant stability. The study protocol was registered in PROSPERO under the registration number CRD42024577122. The PRISMA diagram visually represented the search strategy, screening, and inclusion process. Two reviewers conducted a comprehensive literature search across five databases: PubMed, PubMed Central, Embase, Scopus, and Web of Science. The review synthesized findings from 13 studies; in vitro, in vivo, and clinical studies, highlighting that propolis significantly enhances antibacterial and antifungal activities against pathogens such as *Staphylococcus aureus*, *Candida albicans*, and *Streptococcus mutans*, thereby reducing the risk of peri-implant infections. Additionally, propolis promotes osseointegration by stimulating osteoblast activity and reducing inflammatory cytokine expression, leading to improved bone formation and implant stability. The anti-inflammatory and antioxidant properties of propolis further contribute to a favorable healing environment, enhancing the long-term success of dental implants. The systematic review underscores the potential of propolis as a safe, biocompatible, and effective material for improving dental implant outcomes. However, it also identifies the need for more extensive clinical trials to fully establish standardized protocols for propolis application in implantology. This review provides an overview of propolis’s potential role in dental implants and suggests promising avenues for future research to optimize its benefits in clinical practice.

## 1. Introduction

Dental implants have become a widely accepted and effective solution for the replacement of missing teeth, offering functional and aesthetic benefits that improve patients’ quality of life. However, the success of dental implants is contingent upon several critical factors, including osseointegration, the prevention of peri-implant infections, and the overall stability of the implant within the jawbone. Despite advances in materials and techniques, complications such as infections, inadequate osseointegration, and peri-implantitis remain prevalent, necessitating the exploration of new strategies to enhance implant success rates. One of the primary challenges faced in implantology is the risk of postoperative infections, which can lead to implant failure and additional complications for patients.

Propolis, a natural resinous substance collected by honeybees from various plant sources, has garnered attention for its potential benefits in dental applications. Historically, propolis has been used in traditional medicine for its healing properties, and contemporary studies have confirmed its effectiveness against a broad spectrum of bacterial pathogens [1]. Rich in bioactive compounds such as flavonoids, phenolic acids, and terpenes, propolis exhibits a wide range of biological activities, including antimicrobial, anti-inflammatory, antioxidant, and osteogenic properties [2]. These characteristics make propolis an attractive candidate for enhancing the performance and outcomes of dental implants.

The antibacterial properties of propolis are particularly relevant in the context of dental implants, as infections caused by oral pathogens such as *Staphylococcus aureus*, *Streptococcus mutans*, and *Candida albicans* can lead to implant failure [3,4,5,6]. Studies showed that propolis can inhibit the growth of these pathogens, thereby reducing the risk of peri-implant infections [5,7,8,9]. Additionally, propolis was found to possess antifungal properties, which are crucial for preventing fungal infections in the oral cavity [10].

Osseointegration, the process by which the implant integrates with the surrounding bone, is a critical determinant of implant stability and longevity. Propolis has been shown to promote bone formation and enhance osseointegration by stimulating the activity of osteoblasts and reducing the expression of inflammatory cytokines [11].

Inflammation and oxidative stress are key factors that influence the healing process and the long-term success of dental implants. Propolis is known for its anti-inflammatory and antioxidant properties, which can play a significant role in enhancing the healing process and protecting the implant site from oxidative damage [12].

In recent years, several studies have investigated the incorporation of propolis into various materials and coatings for dental implants [13,14,15,16]. These studies have explored different formulations, concentrations, and methods of application, aiming to optimize the benefits of propolis for implantology.

Given the growing body of evidence supporting the use of propolis in dental implantology, this systematic review aims to evaluate the current research on the effects and benefits of propolis incorporation in dental implants. By analyzing studies from both in vitro and in vivo settings as well as clinical trials, this review seeks to provide a comprehensive understanding of how propolis can enhance dental implant outcomes. The review will also identify gaps in the current knowledge and suggest directions for future research to fully realize the potential of propolis in dental implantology.

This review aims to contribute to the advancement of dental implant technology and improve patient outcomes through the integration of natural, bioactive substances like propolis. By integrating natural, bioactive substances like propolis into dental implant technology, this review aims to enhance both the performance of implants and overall patient outcomes through several key mechanisms: enhanced osseointegration, the anti-inflammatory properties, antimicrobial activity, promotion of soft tissue healing, and biocompatibility.

## 2. Material and Methods

To enhance adherence to systematic review guidelines, the study protocol was registered in PROSPERO CRD42024577122.

### 2.1. Search Strategy

**Figure 1:**
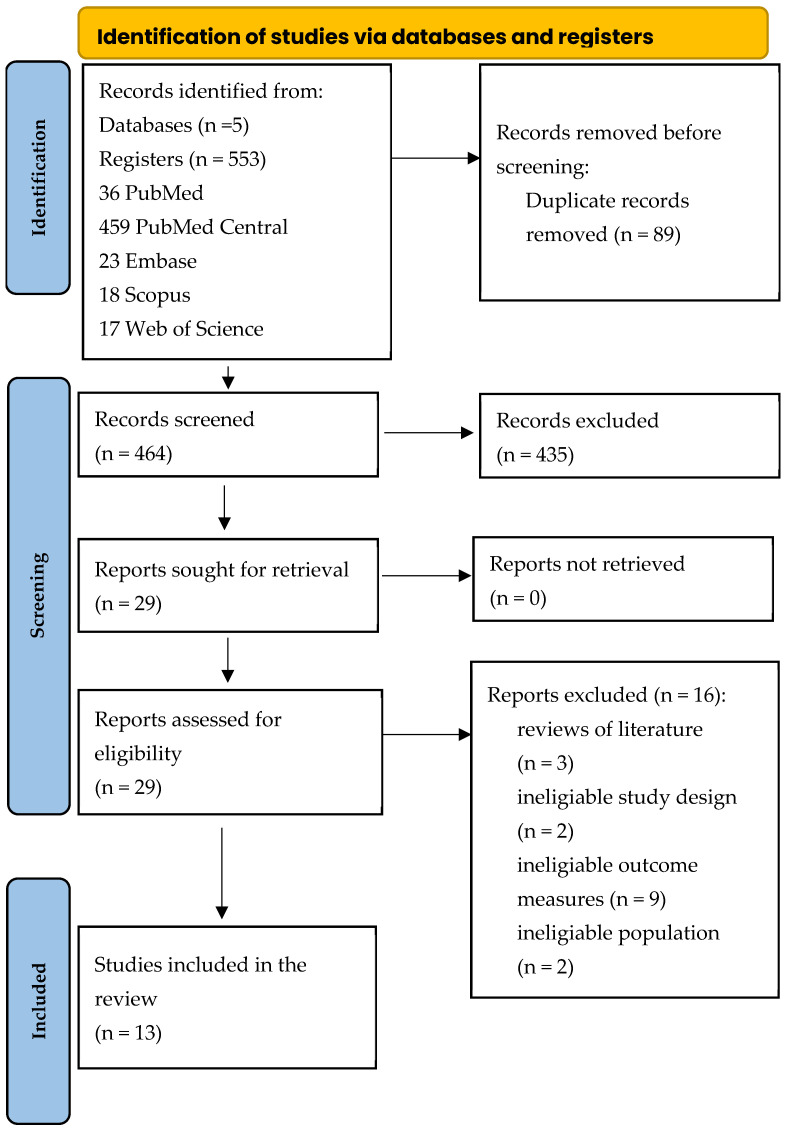
PRISMA flow diagram for the search strategy.

Two independent reviewers conducted a thorough literature search across five databases, including PubMed, PubMed Central, Embase, Scopus, and Web of Science. The search string was carefully crafted to ensure comprehensive coverage of relevant studies, beginning with the formulation for PubMed (“dental” AND “implant*” AND “propolis”) and then tailored for the other databases. Strings for databases: PubMed Central “dental” AND “implant” AND “propolis”, Embase ‘dental’ AND ‘implant’ AND ‘propolis’, Scopus TITLE-ABS-KEY “dental” AND “implant*” AND “propolis”, Web of Science [All Fields] “dental” AND “implant*” AND “propolis”. After completing the extensive search, all identified articles were imported to remove duplicate records. A PRISMA diagram (Figure 1) was created to visually depict the entire search strategy, along with the subsequent screening and inclusion process [17]. PRISMA 2020 manuscript checklist was presented in Supplementary Materials.

Aligned with the PICO framework [18] here is the structured outline for the systematic review: Population (P): studies involving the incorporation of propolis on dental implants or titanium discs; Intervention (I): use of propolis in dental implants or titanium discs; Control (C): dental implants or titanium discs without propolis; Outcome (O): evaluation of the activity of propolis added to dental implants or titanium discs; Research Question: what effect does the incorporation of propolis have on dental implants in in vitro, in vivo animal studies, or clinical research?

The final literature search was completed on 24 August 2024. Notably, no restrictions on publication dates were imposed during the search process, ensuring a comprehensive and unbiased review of relevant publications.

### 2.2. Eligibility Criteria

Based on the systematic review focusing on the effect of propolis incorporation on dental implants, here are the eligibility criteria.

Inclusion Criteria: Study Type: in vitro studies, in vivo animal studies, clinical trials; Intervention: studies that incorporate propolis into dental implants or titanium discs; Control Group: studies must have a control group consisting of dental implants or titanium discs without propolis; Outcome Measures: evaluation of the activity of propolis, including but not limited to antibacterial effects, biocompatibility, osseointegration, and any other relevant biological or mechanical properties; Publication Type: peer-reviewed articles, full-text available, studies published in English.

Exclusion Criteria: Study Type: reviews, commentaries, letters, and editorial articles, case reports and case series without control groups, studies lacking a control group; Intervention: studies that do not focus on the incorporation of propolis in dental implants or titanium discs, studies combining propolis with other substances where the specific effect of propolis cannot be isolated; Outcome Measures: studies not evaluating the activity or effects of propolis on dental implants, studies with unclear or non-specific outcome measures; Publication Type: non-peer-reviewed articles, abstracts without full text available, studies published in languages other than English.

### 2.3. Data Extraction

Following the removal of duplicate publications, the primary author (M.S.-D.) conducted an exhaustive review of the titles and abstracts of the remaining studies. Subsequently, the second author (L.S.-S.) independently assessed all studies to identify those potentially eligible. Full texts of the selected papers were then carefully reviewed, with inclusion or exclusion decisions made based on predefined criteria. Throughout this screening process, both authors worked independently to ensure thoroughness and accuracy. Any uncertainties or ambiguities encountered were resolved through discussions between the two authors, with input from the third author (K.W.) as needed.

To facilitate a comparison of the selected studies, a spreadsheet was created following the Cochrane Collaboration guideline [19]. The level of agreement between the authors was assessed using Cohen’s kappa statistic.

### 2.4. Quality Assessment

The quality of the studies included in the review was evaluated using the Newcastle–Ottawa Scale (NOS) [20]. This tool assigns quality assessment scores ranging from 0 to 9 points, with higher scores indicating superior study quality. The risk of bias in randomized trials (RCTs) was assessed using the Cochrane risk of bias (ROB) 2 tool [21]. Two authors (M.S.-D. and L.S.-S.) independently conducted all assessments, with discrepancies resolved through discussion with a third author (K.W.). To evaluate the level of agreement between the authors, Cohen’s kappa coefficient was calculated, providing a statistical measure of the consistency in the quality assessment process.

## 3. Results

The search strategy identified potential articles from five databases: 36 from PubMed, 459 from PubMed Central, 23 from Embase, 18 from Scopus, and 17 from Web of Science. After removing 89 duplicates, the remaining articles were evaluated. Subsequently, 435 papers were excluded for not meeting the inclusion criteria. From the 29 papers that were eligible, 16 were further excluded due to their lack of relevance to the study’s focus. The final qualitative synthesis comprised 13 papers.

The PRISMA flow diagram (Figure 1) offers a detailed overview of the search and review process, mapping out each stage of the systematic review. Two reviewers demonstrated strong agreement, as reflected by a high Cohen’s kappa coefficient of 0.95, indicating a high level of consensus throughout the evaluation.

Table 1 provides a comprehensive overview of the materials and methods of various studies evaluating the incorporation of propolis in dental implantology, highlighting its potential benefits in enhancing antibacterial properties, promoting bone formation, and improving implant stability. The aggregated results are shown in Table 2.

The systematic review of the effects and benefits of propolis in dental implantology reveals a broad spectrum of positive outcomes. Kehribar et al. [24] demonstrated that incorporating propolis into gel coatings significantly enhanced the antibacterial properties of medical screws, though its effects were limited in control groups. Martorano-Fernandes et al. [9] found that both red propolis and chlorhexidine extract significantly inhibited *C. albicans* proliferation compared to controls (*p* < 0.05). Son et al. [13] reported that dental implants made from poly (L-lactide) (PLA) and poly (ε-caprolactone) (PCL) polymer embedded with propolis-embedded zeolite nanocomposites exhibited effective antibacterial properties with minimal toxicity to normal cells. Somsanith et al. [26] observed significantly greater bone formation and density with propolis-loaded titanium nanotube (TNT) implants in both in vitro studies and in vivo studies. Morawiec et al. [5] in a single-blind, two-group parallel randomized study highlighted the positive biological activity of propolis-containing toothpaste on oral microflora. Additionally, propolis reduced inflammatory cytokines and enhanced collagen fiber expression and osteogenic differentiation proteins.

Further studies reinforced these findings in vivo animal models. Aydin et al. [14] reported significant increases in implant stability in rabbits treated with propolis locally and systemically (*p* < 0.05). Abdulla et al. [22] showed notable bone remodeling and osseointegration in dogs after 180 days, especially in the propolis-coated groups. Aydin et al. [15] observed reduced oxidative stress and increased vitamin D levels (*p* < 0.05) in rabbits treated with propolis. Finally, a study by Krasnikov et al. confirmed that propolis implant coatings exhibited no toxic effects on experimental animals, underscoring their safety and potential as a beneficial adjunct in dental implantology [25]. These cumulative findings suggest that propolis enhances antibacterial efficacy, promotes osseointegration, reduces inflammation, and maintains biocompatibility, making it a promising supplement in dental implant treatments.

### Quality Assessment

Cohen’s kappa coefficient was calculated at 0.94, indicating a high level of agreement between the authors. An evaluation of the quality of the study using the Newcastle–Ottawa scale was collected in Table 3. The quality assessment of the RCTs was shown in Table 4.

## 4. Discussion

The incorporation of propolis into dental implants has garnered significant attention due to its multifaceted benefits, as demonstrated by various in vitro and in vivo studies. This systematic review collates evidence indicating that propolis can significantly enhance the performance and outcomes of dental implants.

The biological activity of propolis frequently varies with propolis sample, dosage, and extraction solvents used. Several studies have highlighted the potent antibacterial and antifungal properties of propolis. Additionally, it was proven that propolis affects the cytoplasmic membrane, inhibits bacterial motility and enzyme activity, exhibits bacteriostatic activity against different bacterial genera and can be bactericidal in high concentrations [28]. However, some data indicate that propolis is active mainly against Gram-positive bacteria and displays much lower activity against the Gram-negative species. Kujumgiev et al. [29] proved that extracts of propolis displayed significant antibacterial activity against *S. aureus* but no active action was noted against *E. coli.* Some authors suggested that the increased resistance of Gram-negative bacteria may be connected to the presence of plasma membrane efflux pumps that would prevent intracellular entry of propolis constituents or promote their extrusion from the cell, or even because propolis contains many plant-derived resin constituents which are secreted to protect plants from mostly Gram-positive pathogens. A number of studies have also been conducted against bacteria of concern in dentistry, which is one of the most important areas of propolis application. Park et al. [30] investigated the use of propolis, collected from various regions of Brazil, for its antibacterial activity and inhibition of glucosyltransferase of *S. mutans*, which is responsible for bacteria activity on the hard surface of teeth. On the basis of their work, it was proven that propolis from Rio Grande do Sul demonstrated both higher antimicrobial activity and inhibition of glucosyltransferase (GTF) activity, which corresponds with different flavonoids in propolis, which in turn correspond with antibacterial and antifungal action. In the context of dental implants, the inhibition of GTF activity by propolis is particularly significant due to its potential to reduce biofilm formation and bacterial colonization on implant surfaces. *Streptococcus mutans*, a primary contributor to biofilm formation on dental surfaces, relies on GTF to produce sticky polysaccharides that allow it to adhere to surfaces and form a cohesive biofilm. Propolis’s ability to inhibit GTF activity directly disrupts this process by reducing the production of these adhesive polysaccharides, thereby hindering the initial stages of biofilm development [31]. Son et al. [13] confirmed that propolis-embedded nanocomposites in dental implants not only provided strong antibacterial efficacy but also exhibited minimal toxicity to normal cells. Kehribar et al. [24] demonstrated that screws coated with propolis exhibited enhanced antibacterial effects against *S. aureus*. Similarly, Martorano-Fernandes et al. [9] found that Brazilian red propolis inhibited the proliferation of *Candida* species more effectively than chlorhexidine. Other data indicate that European propolis has also fungicidal effect against Candida, Microsporum, Mycobacteria, Trichophyton, Fusarium and other dermatophytes [32]. These findings suggest that propolis can significantly reduce the risk of infection, which is a critical factor in the success of dental implants.

Propolis showed a remarkable ability to promote bone formation and enhance osseointegration, which are crucial for the stability and longevity of dental implants. Somsanith et al. [26] observed greater bone formation and density in rats with propolis-loaded TNT implants compared to controls. Aydin et al. [14] reported a significant increase in implant stability in rabbits treated with propolis, both locally and systemically. Abdulla et al. [22] further supported these findings, noting notable osseointegration and bone remodeling in dogs with propolis-coated implants. These studies indicate that propolis can enhance the biological integration of implants with surrounding bone tissue, leading to better clinical outcomes.

The anti-inflammatory properties of propolis are well documented and have been shown to play a significant role in improving implant outcomes [33,34]. Somsanith et al. [26] reported that propolis reduced the expression of inflammatory cytokines such as IL-1β and TNF-α, while enhancing the expression of collagen fibers and osteogenic differentiation proteins. Reducing IL-1β and TNF-α is crucial for enhancing the longevity of dental implants because these cytokines are key mediators in the inflammatory process that can lead to peri-implantitis. Elevated levels of IL-1β have been linked to increased bone resorption and the disruption of the bone-implant interface, ultimately compromising implant stability. Similarly, TNF-α plays a significant role in promoting inflammation and bone loss around implants [35]. Aydin et al. [15] also found that propolis treatment reduced oxidative stress and increased vitamin D levels in rabbits, further contributing to improved implant stability and bone health. Other studies demonstrated that vitamin D deficiency appears to negatively impact the osseointegration of dental implants [36].

In clinical settings, propolis has been shown to positively affect patient outcomes. In both RCTs by González-Serrano et al., 2021 [8] and Morawiec et al. [5] it was demonstrated that gel/toothpaste containing propolis improved the spectrum of oral microflora in patients with dental implants. This suggests that propolis can be beneficial not only as a direct coating for implants but also as an adjunct in oral hygiene products to maintain oral health and prevent peri-implant diseases. Propolis-based toothpastes and mouthwashes have demonstrated favorable outcomes in reducing both gingival inflammation (GI) and plaque index (PI) in patients who did not have dental implants. The natural properties of propolis, which include its anti-inflammatory and antimicrobial effects, contribute significantly to these improvements in oral health. Regular use of these propolis-enriched oral care products helped to maintain healthier gums making propolis a beneficial addition to the daily oral hygiene [37,38,39].

The safety and biocompatibility of propolis are critical for its application in dental implantology. The studies reviewed, including those by Son et al. [13] and Krasnikov et al. [25] indicated that propolis and its composites are non-toxic to normal cells and experimental animals. This underscores the potential of propolis as a safe and effective material for enhancing dental implant performance. While incorporating nano silver into dental implants can offer antimicrobial benefits, it may also present certain toxic effects [40]. In contrast, propolis, a natural substance, tends to have fewer adverse side effects when used in oral care products. Propolis not only provides effective antimicrobial properties but also has low risk of toxicity [41].

Propolis, a resinous substance collected by bees from various plants, has a complex composition that varies widely depending on its botanical source, geographic origin, and environmental conditions. This variability means that propolis samples from different regions may contain distinct proportions of bioactive compounds, such as flavonoids, phenolic acids, terpenes, and other polyphenols, each contributing differently to its biological activity [42,43]. Understanding these compositional differences is crucial when assessing propolis’s therapeutic potential, especially in fields like dentistry where specific biological effects, such as antimicrobial, anti-inflammatory, and regenerative properties are highly desirable. For instance, propolis rich in flavonoids may exhibit stronger antioxidant effects, while samples with higher levels of phenolic acids could show enhanced antibacterial activity [42,44]. Consequently, the efficacy of propolis in dental applications could vary based on these unique chemical profiles, potentially leading to different outcomes in studies conducted in various regions.

The method of extraction, whether aqueous, ethanol, or other types, can also affect the chemical profile and, by extension, the biological activity of propolis. Ethanol extracts, for example, typically yield higher concentrations of certain bioactive compounds than aqueous extracts [45,46]. Highlighting the diversity in propolis’s chemical composition can provide a more accurate understanding of its potential and limitations.

### Limitations and Future Directions

While the results are promising, there are some limitations to the current body of research. Almost all studies were conducted as in vitro or in vivo animal models, and further clinical trials are needed to confirm the efficacy and safety of propolis in human patients. Additionally, standardizing the concentration and formulation of propolis for use in dental implants will be crucial for its widespread adoption. No studies have been identified on the use of orthodontic microimplants with propolis coating. The absence of studies on the use of orthodontic microimplants with propolis coating highlights a significant gap in the current body of research.

## 5. Conclusions

The biological activity of propolis depends on the compounds from the polyphenolic fractions, particularly the flavonoids. The flavonoids isolated from propolis exhibit anti-microbial, antifungal and anti-inflammatory activities. The incorporation of propolis into dental implants offers multiple benefits, including enhanced antibacterial and antifungal properties, improved bone formation and osseointegration due to increased proliferation and differentiation of osteoblasts and reduced inflammation, as well as oxidative stress. These advantages suggest that propolis could be a valuable adjunct in dental implantology, potentially leading to better patient outcomes and higher success rates for dental implants. The use of propolis in dental treatment includes also aphthous ulcers, candidiasis, gingivitis, periodontitis, and pulpitis. Future research should focus on clinical trials and the development of standardized propolis formulations to fully realize its potential in dentistry.

## Figures and Tables

**Table 1:** Summary of studies evaluating the use of propolis.

Authors, Year	Material Evaluated with Propolis and Control Group. Type of Study	Procedure	Time Points Tested	Material Tested
Abdulla et al., 2024 [16]	Four groups: sandblasting with acid etching, sandblasting with Al_2_O_3_, Er-Cr: YSGG laser and propolis coating In vivo animal model	Implant stability quotient	Baseline, after 14 days and after 90 days	Dental titanium implants (Dentium Co., Ltd., Seoul, Republic of Korea)
Abdulla et al., 2024 [22]	Four groups based on surface modifications: sandblasting plus acid etching, sandblasting with Al_2_O_3_, laser and propolis coating In vivo animal model	Osseointegration	Evaluated at 14, 90 and 180 days with radiographs and histological analysis	Dental titanium implants (Dentium Co., Ltd., Seoul, Republic of Korea)
Al-Molla BH et al., 2014 [23]	Propolis-coated implants and control group; implants without propolis In vivo animal model	Osseointegration -osteocalcin and type I collagen markers—immunohistochemical tests	Baseline and at 2, 4, and 6 weeks	Pure titanium implants
Aydin et al., 2017 [14]	Three groups: control, local application of propolis, and systemic application of propolis In vivo animal model	Resonance frequency analysis (RFA) to test stability of implants	Baseline and after 28 days	Dental implants from ADIN Dental Implant Systems (SLA Surface, Toureg-NP, Afula, Israel)
Aydin et al., 2018 [15]	Three groups: local group with propolis solution applied to slots before implant placement; systemic group received daily propolis solution post-implantation; control group implants fixed without propolis In vivo animal model	Blood tests for Vitamin D, phosphor, calcium, and antioxidant enzyme values	Evaluated at 28 days	Dental implants (SLA Surface, Toureg-NP, Afula, Israel)
González-Serrano et al., 2021 [8]	One group of patients treated with a gel containing 2% propolis extract and a control group receiving placebo gel Randomized Controlled Trial	Clinical evaluation (bleeding on probing) and microbiological parameters (CFU counts): *Aggregatibacter actinomycetemcomitans*, *Porphyromonas gingivalis*, *Prevotella intermedia*, *Tannerella forsythia*, *Parvimonas micra*, *Fusobacterium nucleatum*, *Campylobacter rectus*, *Eikenella corrodens*, *Capnocytophaga* sp. and *Actinomyces odontolyticus*	1-month follow-up	Dental implants
Kehribar et al., 2021 [24]	Screws coated with crosslinked polyacrylic acid polymer -Carbopol polymer (10 mg/mL in ethanol) and varying propolis concentrations (2.5%, 5.0%, and 7.5%). Control groups included polymer-only and propolis-only In vitro study	Agar diffusion test *S. aureus* (ATCC 25923)	48 h	Implants (Ti6Al4V ELI) Sandvik Coromant (Sandviken, Sweden)
Krasnikov et al., 2022 [25]	Implants with polymer film (polyazolidine ammonium modified with halogen hydrate ions) and propolis; control group without polymer layer In vivo animal model	Osseointegration	Baseline and after 30 days	Implants (diameter 3.5 mm, length 10 mm)
Martorano-Fernandes et al., 2020 [9]	3% hydroalcoholic extract of Brazilian red propolis. Positive control was chlorhexidine (CHX) at 0.12% and sterile saline solution was the growth control In vitro study	Metabolic activity assay, cell viability (CFU counts) *C. albicans* (ATCC 90028) and *C. glabrata* (ATCC 2001)	96 h after initial adhesion	Pure titanium discs (1.3 cm × 0.2 cm)
Morawiec et al., 2013 [5]	Two groups with either propolis-containing toothpaste or control toothpaste Randomized Controlled Trial	Microbiological tests and evaluation of approximal plaque index, oral hygiene index and sulcus bleeding index	Baseline, after 7 days, and after 8 weeks. Microbiological examination was done at baseline and after 8 weeks	Dental implants supporting prosthetic restorations
Somsanith et al., 2018 [26]	One group was assigned to receive TiO_2_ nanotubes on Ti plate (TNT), the other group was assigned to receive TNT with propolis (PL-TNT-Ti) on Ti plate or/and rod (mini-implant) In vivo animal model	Osseointegration and bone bonding	Evaluated after 1 and 4 weeks	TNT and PL-TNT-Ti implants
Son et al., 2021 [13]	Propolis-embedded zeolite nanocomposites; control group with chlorohexidine (CHX) In vitro study	Agar diffusion test, biofilm inhibition test *C. albicans, S. mutans* and *S. sobrinus*	24-h incubation periods	Dental implants were fabricated using a composite material composed of poly(L-lactide) (PLA)/poly(ε-caprolactone) (PCL) polymer and propolis-embedded zeolite nanocomposites
Srinivas et al., 2022 [27]	The study utilized two solvents for propolis: water and 70% aqueous ethanol In vitro study	Minimum inhibitory concentration, *Aggregatibacter* *Actinomycetemcomitans* (ATCC 43718) total phenolic contents, and total flavonoid content	24-h incubation periods	Implants

CFU, Colony Forming Unit; CHX, chlorhexidine; RFA, Resonance frequency analysis.

**Table 2:** Results of propolis application.

Authors, Year	Conclusion
Abdulla et al., 2024 [16]	Implant stability quotient was related to surface processing and was higher for sandblasting than for other types of treatment.
Abdulla et al., 2024 [22]	After 180 days, osseointegration with notable bone remodeling was particularly evident in the propolis coating group.
Al-Molla BH et al., 2014 [23]	Implants coated with propolis significantly increased osseointegration.
Aydin et al., 2017 [14]	Propolis application resulted in a significant increase in implant stability in both the local and systemic groups compared to the control group (*p* < 0.05).
Aydin et al., 2018 [15]	Propolis reduced oxidative stress. Significant increase in vitamin D level in both propolis groups (*p* < 0.05).
González-Serrano et al., 2021 [8]	After use of propolis gel, 26.1% of patients in the test group achieved complete healing of peri-implant mucositis, compared to no improvement in the control group. The gel with propolis showed antimicrobial effects compared to the control group.
Kehribar et al., 2021 [24]	Adding propolis to the gel coating enhanced the antibacterial properties of the medical screws, while the antibacterial effects were limited for both control groups.
Krasnikov et al., 2022 [25]	Propolis implant coatings have no toxic effects on experimental animals.
Martorano-Fernandes et al., 2020 [9]	Both red propolis and chlorhexidine extract inhibited the proliferation of *C. albicans*, showing statistically significant differences from the control group (*p* < 0.05).
Morawiec et al., 2013 [5]	The study revealed the positive biological activity of toothpaste with propolis on the spectrum of oral microflora.
Somsanith et al., 2018 [26]	Bone formation and bone density were significantly greater with the propolis-loaded TNT implants compared to the drug-free TNT implants. Propolis reduced the expression of inflammatory cytokines like IL-1β and TNF-α and enhanced the expression of collagen fibers and osteogenic differentiation proteins.
Son et al., 2021 [13]	Dental implants made from poly (L-lactide) (PLA)/poly(ε-caprolactone) (PCL) polymer with propolis-embedded zeolite nanocomposites exhibit antibacterial efficacy and negligible toxicity to normal cells.
Srinivas et al., 2022 [27]	Propolis extracted using water as the solvent demonstrated a superior minimum inhibitory concentration and exhibited higher total phenolic content and total flavonoid content compared to propolis extracted with alcohol as the solvent. Propolis was effective against *Aggregatibacter actinomycetemcomitans*, suggesting that it may be used in the treatment of peri-implantitis.

**Table 3:** Evaluation of the quality of the study conducted using the Newcastle–Ottawa scale.

Authors and Year	Selection	Comparability	Outcome	Total Score
Abdulla et al., 2024 [16]	***	**	**	7
Abdulla et al., 2024 [22]	***	**	**	7
Al-Molla BH et al., 2014 [23]	***	**	**	7
Aydin et al., 2017 [14]	***	**	**	7
Aydin et al., 2018 [15]	***	**	***	8
Kehribar et al., 2021 [24]	**	**	**	6
Krasnikov et al., 2022 [25]	**	**	**	6
Martorano-Fernandes et al., 2020 [9]	***	**	**	7
Somsanith et al., 2018 [26]	***	**	**	7
Son et al., 2021 [13]	***	**	**	7
Srinivas et al., 2022 [27]	***	**	**	7

The NOS application consists of assigning a maximum of one point (one star (*)) for each numbered item within the selection and outcome categories, except for the item comparability, in which a maximum of two stars (**) can be given. Therefore, according to NOS protocol, papers are awarded stars for three different criteria, i.e., selection (worth a maximum of 4 stars (****)), comparability (a maximum of 2 stars (**)), and outcome (a maximum of 3 stars (***)) to earn a maximum score of 9 stars.

**Table 4:** Evaluation of the quality of the RCTs.

Author, Year	Randomization Process	Deviations from the Intended Interventions	Missing Outcome Data	Measurement of the Outcome	Selection of the Reported Results	Overall
González-Serrano et al., 2021 [8]	Low risk	Low risk	Some concerns	Low risk	Low risk	Low risk
Morawiec et al., 2013 [5]	Low risk	Low risk	Some concerns	Low risk	Low risk	Low risk

## Data Availability

The original contributions presented in the study are included in the article, further inquiries can be directed to the corresponding author.

## Supplementary Materials

The following supporting information can be downloaded at: https://www.mdpi.com/article/10.3390/jfb15110339/s1, Supplementary file: PRISMA 2020 manuscript checklist.
